# M1 macrophage features in severe *Plasmodium falciparum* malaria patients with pulmonary oedema

**DOI:** 10.1186/s12936-020-03254-0

**Published:** 2020-05-15

**Authors:** Aekkarin Klinkhamhom, Supattra Glaharn, Charit Srisook, Sumate Ampawong, Srivicha Krudsood, Stephen A. Ward, Parnpen Viriyavejakul

**Affiliations:** 1 Department of Tropical Pathology, Faculty of Tropical Medicine, Mahidol University, 420/6 Rajvithi Road, Bangkok, 10400 Thailand; 2 Department of Tropical Hygiene, Faculty of Tropical Medicine, Mahidol University, 420/6 Rajvithi Road, Bangkok, 10400 Thailand; 3 Research Centre for Drugs and Diagnostics, Liverpool School of Tropical Medicine, Liverpool, L3 5QA UK

**Keywords:** Lung macrophages, M1, M2, *Plasmodium falciparum*, Pulmonary oedema, Malarial pigment, Haemozoin

## Abstract

**Background:**

Pulmonary oedema (PE) is a serious complication of *Plasmodium falciparum* malaria which can lead to acute lung injury in severe cases. Lung macrophages are activated during malaria infection due to a complex host-immune response. The molecular basis for macrophage polarization is still unclear but understanding the predominant subtypes could lead to new therapeutic strategies where the diseases present with lung involvement. The present study was designed to study the polarization of lung macrophages, as M1 or M2 macrophages, in the lungs of severe *P. falciparum* malaria patients, with and without evidence of PE.

**Methods:**

Lung tissue samples, taken from patients who died from severe *P. falciparum* malaria, were categorized into severe malaria with PE and without PE (non-PE). Expression of surface markers (CD68+, all macrophages; CD40+, M1 macrophage; and CD163+, M2 macrophage) on activated lung macrophages was used to quantify M1/M2 macrophage subtypes.

**Results:**

Lung injury was demonstrated in malaria patients with PE. The expression of CD40 (M1 macrophage) was prominent in the group of severe *P. falciparum* malaria patients with PE (63.44 ± 1.98%), compared to non-PE group (53.22 ± 3.85%*, p* < 0.05), whereas there was no difference observed for CD163 (M2 macrophage) between PE and non-PE groups.

**Conclusions:**

The study demonstrates M1 polarization in lung tissues from severe *P. falciparum* malaria infections with PE. Understanding the nature of macrophage characterization in malaria infection may provide new insights into therapeutic approaches that could be deployed to reduce lung damage in severe *P. falciparum* malaria.

## Background

Pulmonary oedema (PE) is one of the major complications and therapeutic challenges in severe *Plasmodium falciparum* malaria. This condition is associated with acute lung injury (ALI) and acute respiratory distress syndrome (ARDS) [1]. The incidence of ARDS in adults with severe *P. falciparum* malaria ranges from 7.6 to 26.2% [2–5], with the mortality rate of 52.2–89.0% [2, 5, 6]. In addition, PE occurs in approximately 10–21% [1, 7, 8]. In *P. falciparum* malaria, PE is associated with inflammatory infiltrates consisting of mainly mononuclear cells, haemozoin deposit, the accumulation of macrophages, as well as parasite sequestration [9]. Recruitment of macrophages to the lung signifies an important immune response in the pathogenesis of ALI/ARDS [10]. Activated macrophages have been described as two functional subsets, namely classically activated macrophages (M1) and alternatively activated macrophages (M2) [11, 12]. M1 macrophages are considered as pro-inflammatory macrophages that produce pro-inflammatory cytokines, such as tumour necrosis factor (TNF), interleukin-6 (IL-6) and other mediators. M2 macrophages are typically anti-inflammatory macrophages and produce anti-inflammatory cytokines, such as IL-10 and secrete growth factor such as transforming growth factor beta 1 (TGF-β1) for tissue repair [12].

The polarization of M1 and M2 macrophages are important for disease regulation. Previous studies have documented that activated macrophages are prominent in lung infection with bacteria [13] and viruses [14, 15] as well as in lungs from smokers and chronic obstructive pulmonary disease (COPD) [16]. The main purpose of this study was to investigate the status of lung macrophages, as to M1/M2 subtypes in severe *P. falciparum* malaria patients with and without PE.

## Methods

### Tissue specimens

Embedded human lung tissues from severe *P. falciparum* malaria infected patients and non-infected controls were retrieved from the Department of Tropical Pathology, Faculty of Tropical Medicine, Mahidol University, Bangkok, Thailand. Twenty-four cases from severe *P. falciparum* malaria were originally available for evaluation. Lung tissues from seven cases were inadequate for further studies. Of the 17 cases, they were classified into severe *P. falciparum* malaria with PE (n = 9) and non-PE (n = 8), according to histopathological findings. In addition, there were 6 normal lung tissues from control cases. Lung tissues were embedded and prepared for histopathological evaluation. Based on histopathological findings of oedematous fluid in the lung, lung tissues were divided into severe *P. falciparum* malaria with PE, non-PE and control lung tissues. Normal control lung tissues were obtained from patients who died from accidents, and showed no pathological changes in the lungs. The study protocol was reviewed and ethical clearance was obtained from the Ethics Committee of Faculty of Tropical Medicine, Mahidol University (MUTM 2017-054-01).

### Histopathology and evaluation

Lung tissues were re-embedded with new paraffin medium, sectioned at 4 μm in thickness and routinely stained with haematoxylin and eosin (H&E). The pathological changes of lung tissues were interpreted based on eight histological criteria in twenty low power fields (LPF) (200×) per slide, namely septal congestion, alveolar haemorrhage, alveolar oedema, hyaline membrane formation, parasitized red blood cell (PRBC) sequestration, malarial pigment, lung macrophages and infiltration of inflammatory cells [17]. Each variable was graded on a scale based on percentage of severity based on a previous study with modifications, as follows: no injury = 0, injury ≤ 25%/HPF = 1, injury > 25% and ≤ 50%/HPF = 2, injury > 50% and ≤ 75%/HPF = 3, and injury > 75%/HPF = 4 [18]. Lung macrophages and white blood cells (WBC) were quantified and graded as follows: no cell = 0, cells ≤ 25/HPF = 1, cells > 25 and ≤ 50/HPF = 2, cells > 50 and ≤ 75/HPF = 3, and cells > 75/HPF = 4. Subsequently, a lung injury score ranging from 0 to 32 points was calculated by adding the sum of each variable to determine the overall histopathological changes in lung tissues from malaria patients with *P. falciparum*. A score of 0 means absence of histopathological changes while a score of 32 signified the most severe histopathological changes. All histopathological parameters were examined in a blinded manner, without prior knowledge of the patients’ clinical status. Two independent assessors (AK, PV) evaluated the histopathological specimens. If there were discrepancies of more than 2 grading variations, a third person (SG/CS) was requested to score the histopathological changes and the mean readings of the three assessors were taken.

### Immunohistochemical evaluation of lung macrophages

The expressions of macrophage surface markers (CD68, CD40, and CD163) were detected by immunohistochemical staining [19, 20]. Lung sections of 4 μm thickness were placed on adhesive slides coated with poly-l-lysine and de-paraffinized through a series of xylene and re-hydrated through graded alcohol solutions. Antigen retrieval from lung tissues was performed by a microwave technique with 0.01 mol/L citrate buffer at pH 6.0 for 20 min. To reduce endogenous peroxidase activity, tissue sections were incubated with 1% H_2_O_2_ in PBS for 30 min at room temperature (RT) and rinsed in running tap water for 10 min. Lung sections were treated with normal goat serum for 30 min at RT to reduced non-specific background. To detect the surface makers on macrophages, lung sections were incubated with primary antibody: monoclonal mouse anti-human CD68 (1:100 optimal dilution, DakoCytomation^®^; Denmark), mouse monoclonal antibody CD163 (1:200 optimal dilution, Novocastra™; Leica Biosystems Newcastle Ltd, UK), and rabbit polyclonal antibody CD40 (1:200 optimal dilution, Santa Cruz Biotechnology Inc., USA) in a moisture chamber overnight at 4 °C. Next day, tissue sections were extensively washed with PBS, and incubated with secondary antibody for 30 min at RT, then incubated with avidin-biotin complex (ABC) conjugated with horseradish peroxidase (HRP) (VECTASTAIN^®^ ABC Kits; Vector Laboratories, USA) for 30 min at RT. After the removal of non-reacted secondary antibodies, all sections were incubated with DAB (3,3′-diaminobenzidine) Peroxidase Substrate Kit (Vector Laboratories, USA) for 3 min. Finally, sections were counterstained with Mayer’s haematoxylin, mounted with a coverslip and evaluated under a light microscope. Spleen was used as a positive control for CD68, CD40 and CD163 [21]. The anti-CD68 within the tissue sections were used as positive internal controls. Positive cells stained brown pattern with different degrees of immunoreactive intensity.

### Evaluation of immunohistochemistry staining

Quantitative assessments of macrophage subtypes were based on the number of positive stained cells for CD68, CD40 and CD163. Macrophages were evaluated randomly in twenty fields per slide, in lung tissues and within the septal area under high magnification (400×). The numbers of surface markers specific for each macrophage subtype were counted and expressed as percentage of positive cells. The intensity of positive stained cells was graded as follows: 0 = negative; 1 = weak staining; 2 = moderate staining; 3 = strong staining. The total score (TS) for this immunohistochemical study was determined by obtaining the product of the percentage of positive cells and the intensity of the staining [22, 23]. The sections were examined in a blinded manner, without prior knowledge of the patients, clinical status.

### Statistical analysis

All quantitative data were expressed as mean ± standard error of the mean (SEM). Statistical analysis was performed using SPSS version 18.0 software (SPSS, USA). Data was tested with Kolmogorov–Smirnov test for normality of distribution. Mann–Whitney *U* test was used to analyse differences in clinical data, clinical complications and histopathological criteria between non-PE, PE and control groups. Difference in CD40 (M1) and CD163 (M2) expression between groups was interpreted by one-way analysis of variance. The correlations between total score of M1 expression and histological/clinical data were analysed by Spearman’s correlation. Statistical differences at *p* < 0.05 were considered to be statistically significant.

## Results

### Summary of clinical and laboratory data from malaria patients

The demographic data from severe *P. falciparum* malaria patients is documented in Table 1. Significant differences were observed in levels of haematocrit (*p* = 0.032) and creatinine (*p* = 0.023) between non-PE and PE groups. No significant difference in age, blood urea nitrogen (BUN), alanine transaminase (ALT), aspartate aminotransferase (AST), alkaline phosphatase (ALP), albumin, globulin, total bilirubin, direct bilirubin, WBC counts, parasitaemia and days of fever (all *p* > 0.05) was observed. Clinical adult respiratory distress syndrome (ARDS) and acute kidney injury were two complications that showed significant difference between non-PE and PE groups (Additional file 1: Table S1). The causes of death were attributed mainly to cerebral malaria based on clinical manifestations and histopathological findings. Usually patients died of multiple complications of *P. falciparum* malaria. No difference in other clinical complications of severe malaria (cerebral malaria, jaundice/hepatic dysfunction, severe anaemia, severe metabolic acidosis, hypoglycaemia, shock and disseminated intravascular coagulopathy) was observed between the two groups (Additional file 1: Table S1).

**Table 1:** Clinical data of the *P. falciparum* malaria patients

Parameters	Severe malaria patients (mean ± SEM)		*p*-values
	Non-PE (n = 12)	PE (n = 12)	
Age (years)	24.00 ± 5.09	27.00 ± 2.53	0.600
Haematocrit (%)	32.20 ± 2.91	24.00 ± 1.53	0.032*
Blood urea nitrogen (BUN) (mg/dl)	21.30 ± 3.16	51.07 ± 13.07	0.072
Creatinine (mg/dl)	1.45 ± 0.29	3.28 ± 0.72	0.023*
Alanine transaminase (ALT) (U/l)	179.33 ± 150.42	142.75 ± 10.76	0.355
Aspartate aminotransferase (AST) (U/l)	269.75 ± 193.05	133.25 ± 13.39	0.257
Alkaline phosphatase (ALP) (U/l)	75.33 ± 36.75	55.15 ± 0.57	0.133
Albumin (g/l)	2.93 ± 0.36	2.50 ± 0.26	0.375
Globulin (g/dl)	2.75 ± 0.19	3.15 ± 0.38	0.384
Total bilirubin (mg/dl)	3.33 ± 1.75	6.87 ± 3.82	0.467
Direct bilirubin (mg/dl)	8.09 ± 4.13	12.08 ± 6.21	0.630
White blood cell (WBC) (/µl)	25,291 ± 15,400	25,200 ± 12,947	0.101
Parasitaemia/µl on admission	401.00 ± 369.95	93.50 ± 56.28	0.485
Days of fever	3.25 ± 0.52	4.33 ± 0.92	0.418

* Significant difference of *p *< 0.05

### Histopathological changes in the lungs of *P. falciparum* malaria patients

**Fig. 1:**
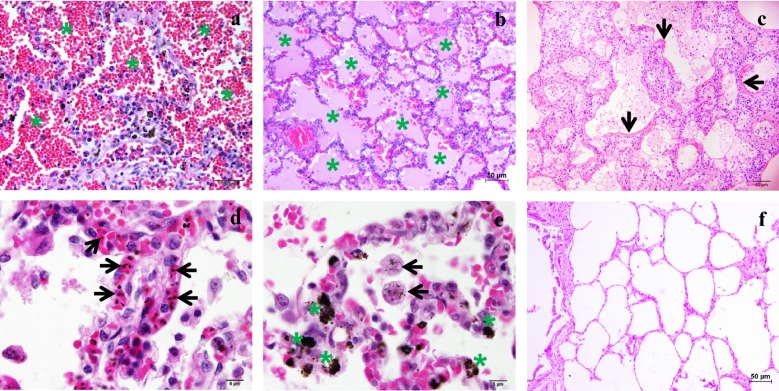
Histopathological changes of lungs in *P. falciparum* malaria: **a** alveolar congestion and haemorrhage (asterisks), **b** alveolar oedema (asterisks), **c** hyaline membrane formation in alveolar wall (arrows), **d** sequestration of PRBCs (arrows), **e** malarial pigment laden macrophages in septal area (asterisks) and in alveolar spaces (arrows), **f** normal lung from control sample (Images **a** ×200, **b**, **c** and **f** ×100, **d** and **e** ×400)

**Fig. 2:**
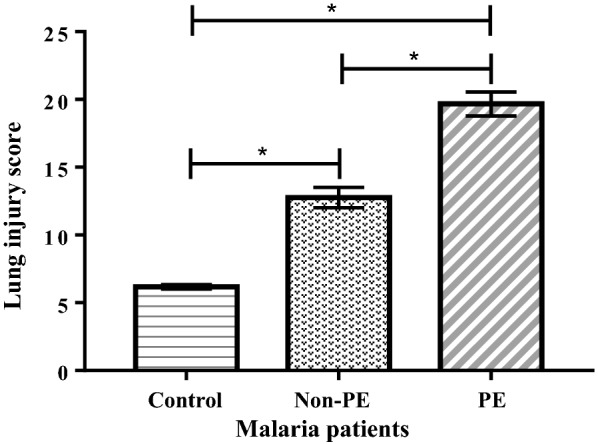
Lung injury score in malaria patients. A significant difference was observed between PE and non-PE (*p* < 0.05). Data presented as mean ± SEM

Common histopathological changes in the lungs of severe *P. falciparum* malaria included the presence of septal congestion and alveolar haemorrhage (Fig. 1a), alveolar oedema (Fig. 1b), hyaline membrane formation (Fig. 1c), PRBC sequestration in pulmonary capillaries (Fig. 1d), malarial pigment and an increase in the number of macrophages in alveolar spaces and septal area (interstitial area) (Fig. 1e) (all *p* < 0.05, compared to control lung, Fig. 1f). Mixed inflammatory cells are occasionally seen within the alveoli. Comparing non-PE and PE groups, significant difference in histological findings were noted in the presence of alveolar haemorrhage, alveolar oedema, accumulation of malarial pigment and the number of lung macrophages (all *p* < 0.05). The calculated lung injury score (based on histopathological criteria) was significantly higher in both the non-PE and the PE group, compared to control group (all *p* = 0.001, Additional file 2: Table S2). Moreover, the lung injury score was significantly higher in PE group (19.67 ± 0.88), compared to non-PE group (12.75 ± 0.75, *p* = 0.001) (Fig. 2). Other non-lung histopathological findings are listed in Table 2. Acute tubular necrosis was more prevalent in PE group compared to non- PE group (*p* = 0.038).

**Table 2:** Non-lung histopathological findings of *P. falciparum* malaria patients

Non-lung histopathological findings	Non-PE	PE	*p*-values
Brain	(n = 10)	(n = 12)	
PRBC sequestration	7	9	0.798
Petechial/ring haemorrhages	8	9	0.785
Heart	(n = 10)	(n = 9)	
PRBC sequestration	7	6	0.879
Liver	(n = 12)	(n = 11)	
Kupffer cell proliferation	12	11	1.000
Haemozoin deposit	10	10	0.598
PRBC sequestration	9	10	0.325
Kidney	(n = 10)	(n = 9)	
Mesangial proliferations	5	5	0.814
Protein deposition in Bowman’s space	1	3	0.225
PRBC sequestration	7	8	0.326
Haemozoin deposit	8	8	0.606
Acute tubular necrosis	1	5	0.038*

*Significant difference of *p *< 0.05

### Immunohistochemistry study of M1/M2

#### Expression of CD68

**Fig. 3:**
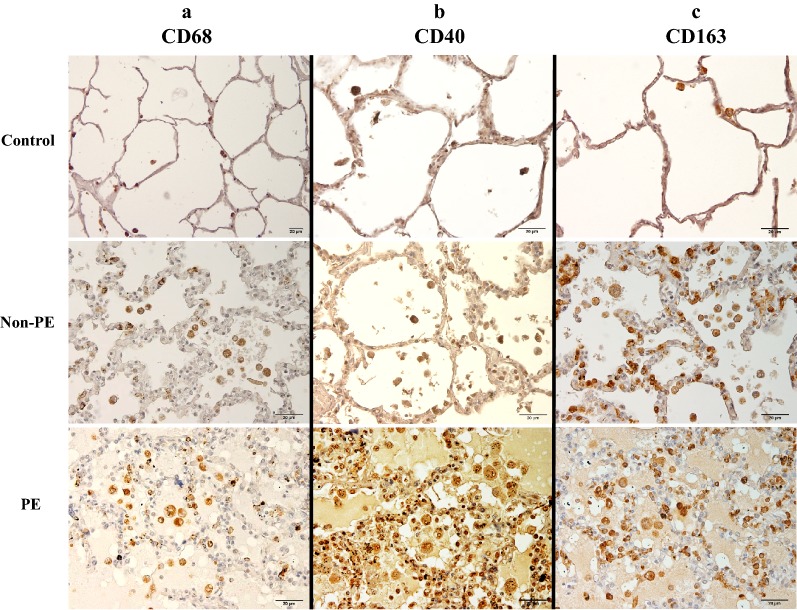
The immunohistochemical staining of lung macrophages in *P. falciparum* malaria. Expressions of CD68 (**a**), CD40 (**b**), and CD163 (**c**), were demonstrated in control, non-PE and PE groups. CD68 expression showed fine brown color in the cytoplasmic granules. CD40 expression was evident on cell membrane while CD163 positive cells showed fine brown color in the cytoplasm of lung macrophages. (All images: ×400, Avidin–Biotin–peroxidase complex technique)

CD68 expressing cells were detected as fine brown color in the cytoplasm of lung macrophages in both the septal area and within the alveoli (Fig. 3a). The number of macrophage positive cells was significantly increased in PE group (49.23 ± 5.16/HPF) compared to the non-PE group (31.22 ± 3.81/HPF, *p* = 0.016) and the normal control group (6.40 ± 1.39/HPF, *p* = 0.001). In septal areas, no significant difference in CD68 positive cells was noted between PE (36.64 ± 4.50/HPF), and non-PE groups (24.41 ± 3.40/HPF, *p* = 0.118). However, both malaria groups showed significantly higher CD68 positive cells compared to the control group (3.58 ± 0.99/HPF, *p* < 0.05).

#### Expression of CD40

**Fig. 4:**
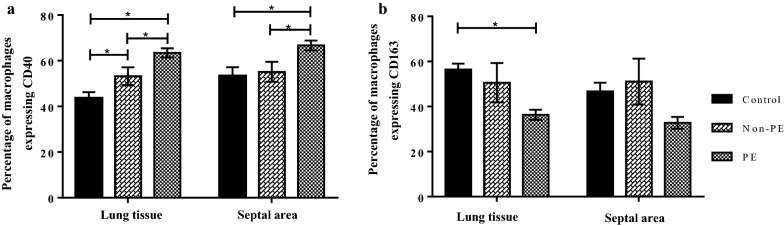
Comparative mean percentages of lung macrophage positive cells for CD 40 (**a**) and CD 163 (**b**) of control, non-PE and PE groups

CD40 was used to detect activated M1 subtype lung macrophages. Positive cells expressed a fine brown color on the cell membrane of macrophages. The relative expression of CD40 was prominent in the group of severe *P. falciparum* malaria with PE (63.44 ± 1.98%) compared to non-PE (53.22 ± 3.85%*, p *= 0.018) and the control group (43.59 ± 2.61%, *p *< 0.01) (Figs. 3b, 4a). Macrophages expressing CD40 in the alveolar septal area between PE and non-PE groups showed similar trends as in the lung tissues, as depicted in Fig. 4a.

#### Expression CD163

CD163 was used as a marker for the M2 subtype. Positive cells express a fine brown color in the cytoplasm of macrophages. Lung macrophages in severe *P. falciparum* malaria patients with PE showed a relative decrease CD163 expression, compared to the control group (*p *< 0.024). No significant difference in the mean percentage of CD163 positive cells was observed between PE and non-PE groups (*p* = 0.075), and between non-PE and control groups (*p* = 0.493). In addition, no significant difference in CD163 expression in the septal area was noted among three groups (*p* > 0.05) (Figs. 3c, 4b).

### Correlations between M1 total score and histopathological changes

**Fig. 5:**
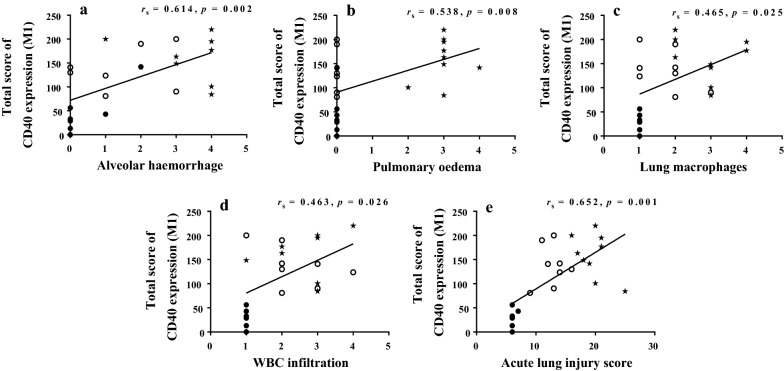
Correlations between total score of CD40 expression (M1) and histopathological changes (**a**–**e**). Total score = the percentage of positive cells x staining intensity (filled circle = control, open circle = non-PE, and filled star = PE subjects)

There was a significant positive correlation between the total score of CD40 expression (M1) and alveolar haemorrhage (*r*_s_ = 0.614, *p *= 0.002), alveolar oedema (*r*_s_ = 0.538, *p *= 0.008), the number of lung macrophages (*r*_s_ = 0.465, *p *= 0.025), WBC infiltration (*r*_s_ = 0.463, *p *= 0.026) and acute lung injury score (*r*_s_ = 0.652, *p *= 0.001) (Fig. 5a–e). Due to the distribution of variables, correlation analysis was unsuitable to perform for septal congestion, hyaline membrane formation, PRBC sequestration and presence of malaria pigments.

## Discussion

Acute lung injury (ALI) was demonstrated in severe *P. falciparum* malaria patients, with more severe changes in patients with pulmonary oedema (PE). The pathological findings of septal congestion, alveolar haemorrhage, alveolar oedema, hyaline membrane formation, malarial pigment, increase number of lung macrophages (both alveolar and interstitial), and white blood cells are commonly seen in lung tissues of fatal *P. falciparum* malaria, in addition to the presence of PRBCs.

PE is a common finding in ALI and can cause the lungs to develop into acute respiratory distress syndrome (ARDS) [24]. The activation of lung macrophages and release of cytokines are important mechanisms contributing to lung damage in patients with ARDS [19, 25]. Under various triggers, lung macrophages can be polarized into either classically activated (or M1) or alternatively activated (or M2) subtypes [26]. M1 macrophages can be stimulated by interferon (IFN)-γ, lipopolysaccharide (LPS) and granulocyte–macrophage colony stimulating factor, causing upregulation of genes involved in pathogen clearance and an inflammatory response. M2 macrophages, on the other hand, are induced by interleukin (IL)-4 and IL 13 and cause upregulation of genes involved in wound healing, phagocytic activity and an anti-inflammatory responses [26]. The main secreting cytokines from M1 macrophages which contribute to ALI include tumour necrosis factor (TNF), IL-1β and reactive oxygen species [27]. In malaria, an essential factor that can contribute to the pathogenesis of lung injury is haemozoin pigment present in the lung tissue. A previous study in an animal model reported that haemozoin pigment can activate pro-inflammatory mediators and is related to the occurrence of ARDS in mice [28]. The subsequent influx of inflammatory cells into the lung tissue damages endothelial cells and results in increased vascular permeability [29]. It is possible that haemozoin activated pro-inflammatory chemokines (i.e., IFN-γ, CXCL 10), and cytokines (i.e., TNF, IL-6) [30] can contribute to M1 polarization in malaria, resulting in ALI/ARDS. Consequently, pathological changes of PE, pulmonary haemorrhage, further recruitment of inflammatory cells and hyaline membrane formation may occur. In addition, macrophages can adhere and interact with the epithelium of alveoli resulting in an increase in cytosolic calcium levels which can lead to apoptosis of epithelial cells and the release of TNF. This mechanism can further damage the pulmonary barrier resulting in PE [25].

Malaria infection triggers recruitment of resident macrophages to the lungs as evident by an increase in CD68 expressing cells. Results demonstrated the accumulation of M1 macrophages in the lung of severe *P. falciparum* malaria patients, especially in the PE group as demonstrated by an increase in CD40 expressing cells. Similar findings were documented in mice lung infected with *Escherichia coli* [31], in lung-induced chronic obstructive pulmonary disease (COPD) [16], and in viral infection in mice [14]. In addition to M1 being associated with accumulation of haemozoin and the occurrence of acute lung injury, polarization of macrophages to the M1 subtype is responsible for controlling infectious diseases during the acute phase. In influenza virus infection, lung macrophages can polarize to M1 in the early stage and later present the M2b phenotype. Functionally, M1 macrophages are characterized by enhanced endocytic functions and enhanced ability to kill intracellular pathogens [32]. In *Listeria monocytogenes* infection, M1 macrophages were reported to be activated. These macrophages help prevent bacterial phagosome escape and stimulate intracellular killing of bacteria [31].

From this study, M1 (CD40) macrophages predominate in the PE group and correlate directly with lung pathology, providing evidence for M1 associated damaged lung tissue in severe *P. falciparum* malaria. M2 polarization (CD163) however, was similar in both the PE and non-PE group. It would be optimal to simultaneously analyse the expressions of CD68, CD40 and CD163 in lung macrophages by incorporating these three antigens and their co-expression on a single lung tissue. Thus, avoiding certain non-specific staining that could interfere with the analysis. In addition to the limitation on staining technique used, it is important to note that evaluating antigen expressions in the lung tissues from different groups could not be entirely blinded. Although, assessors were unaware of the clinical groups and were provided with blind-coded lung tissue slides; the presence of fluid in the alveoli could somehow create an unavoidable bias during microscopic evaluation.

Previous reports documented an increase CD163 levels in uncomplicated malaria patients as compared to severe malarial anaemia and cerebral malaria patients. This phenomenon is suggestive of a higher-level anti-inflammatory response in uncomplicated malaria in order to avoid disease complications [33]. Since lung samples from this study was available only once, at autopsy, no data on sequential M1/M2 shifts between early and late infection in human malaria is possible. Theoretically, at a later stage of malaria infection, after pathogenic factors are eliminated through therapeutic anti-malarial treatment and immune clearance of malaria parasites, lung macrophages may shift from a M1 phenotype to the anti-inflammatory M2 phenotype. The switching of macrophage polarity has been associated with transcriptional control [34]. M2 macrophages have an important role in lung tissue repair by limiting the levels of proinflammatory cytokines in the cellular space and producing anti-inflammatory cytokines such as IL-10 and IL-1 receptor antagonist [10]. M2 macrophages also remove necrotic cells and debris through phagocytic activity, which can be demonstrated in the malaria lung tissues as lung macrophages engulf haemozoin and fragmented PRBCs.

## Conclusions

Pulmonary macrophages are believed to be an important factor in causing lung pathology in malaria. The study showed M1 macrophage activation in ALI in severe *P. falciparum* malaria, which indicates disease severity. As M1 polarization prevails, more lung damage can occur. Further investigation on the pathways of the malaria-induced M1 phenotype is necessary to identify specific activators for M1 polarization that could be attenuated and factors that promote the anti-inflammatory M2 phenotype, in order to suppress the inflammation and improve clinical ALI in severe malaria patients.

## Supplementary information

**Additional file 1: Table S1.** Clinical complications of severe *P. falciparum* malaria patients in non-PE and PE groups.

**Additional file 2: Table S2.** Histopathological criteria for acute lung injury in severe *P. falciparum* malaria patients in non-PE, PE and control groups. The reported scores are based on the graded percentage of severity.

## Data Availability

All data generated or analysed during this study are included in this published article.

## Abbreviations

ABC: Avidin–biotin complex
ALI: Acute lung injury
ALP: Alkaline phosphatase
ALT: Alanine transaminase
ARDS: Adult respiratory distress syndrome
AST: Aspartate aminotransferase
BUN: Blood urea nitrogen
CD: Cluster differentiation
COPD: Chronic obstructive pulmonary disease
CXCL: C-X-C motif ligand
DAB: 3,3′-diaminobenzidine
H&E: Haematoxylin and eosin
HPF: High power field
HRP: Horseradish peroxidase
IFN: Interferon
IL: Interleukin
LPF: Low power field
LPS: Lipopolysaccharide
M1: Classically activated macrophages
M2: Alternatively activated macrophages
*p*: Probability value
PBS: Phosphate-buffered saline
PE: Pulmonary oedema
PRBC: Parasitized red blood cells
*r*_s_: Spearman correlation coefficient
RT: Room temperature
SD: Standard deviation
SEM: Standard error of the mean
SPSS: Statistical package for the social sciences
TGF-β1: Transforming growth factor beta 1
TNF: Tumour necrosis factor
TS: Total score
WBC: White blood cells
