# Supplementary material for: M1 macrophage features in severe Plasmodium falciparum malaria patients with pulmonary oedema
Source: Malar J. 2020 May 15;19:182. doi: 10.1186/s12936-020-03254-0 (PMC7226720; doi:10.1186/s12936-020-03254-0)
Supplement: Supplementary file 1 — Additional file 1: Table S1. Clinical complications of severe P. falciparum malaria patients in non-PE and PE groups. [file 12936_2020_3254_MOESM1_ESM.doc]

M1 macrophage features in severe *Plasmodium falciparum* malaria patients with pulmonary oedema

**Additional data**

**Table S1. Clinical complications of severe *P. falciparum* malaria patients in non-PE and PE groups**

| **Clinical complications** | **Non- PE (n=12)** | **PE (n=12)** | ***p*-values** |
| --- | --- | --- | --- |
| Cerebral malaria | 10 | 11 | 0.468 |
| Adult respiratory distress syndrome | 1 | 6 | 0.028* |
| Jaundice/ hepatic dysfunction | 3 | 6 | 0.216 |
| Severe anaemia | 0 | 3 | 0.070 |
| Severe metabolic acidosis | 0 | 2 | 0.148 |
| Hypoglycaemia | 0 | 1 | 0.317 |
| Shock | 2 | 0 | 0.148 |
| Disseminated intravascular coagulopathy | 1 | 0 | 0.317 |
| Acute kidney injury | 0 | 4 | 0.030* |

*****Significant difference of *p* < 0.05
