# Supplementary material for: M1 macrophage features in severe Plasmodium falciparum malaria patients with pulmonary oedema
Source: Malar J. 2020 May 15;19:182. doi: 10.1186/s12936-020-03254-0 (PMC7226720; doi:10.1186/s12936-020-03254-0)
Supplement: Supplementary file 2 — Additional file 2: Table S2. Histopathological criteria for acute lung injury in severe P. falciparum malaria patients in non-PE, PE and control groups. The reported scores are based on the graded percentage of severity. [file 12936_2020_3254_MOESM2_ESM.doc]

M1 macrophage features in severe *Plasmodium falciparum* malaria patients with pulmonary oedema

**Additional data**

**Table S2. Histopathological criteria for acute lung injury in severe *P. falciparum* malaria patients in non-PE, PE and control groups**. **The reported scores are based on the** **graded percentage of severity**.

| **Histopathological criteria** | **Non- PE (n=8)** | **PE**  **(n=9)** | **Control (n=6)** | ***p*-values*** |
| --- | --- | --- | --- | --- |
| Septal congestion | 4.00±0.00 | 4.00±0.00 | 4.00±0.00 | 1.000 |
| Alveolar haemorrhage | 1.50±0.42 | 3.22±0.36 | 0.17±0.17 | 0.010** |
| Alveolar oedema | 0 | 3.00±0.17 | 0 | 0.000** |
| Hyaline membrane formation | 0.50±0.50 | 0 | 0 | 0.392 |
| PRBCs sequestration | 1.00±0.66 | 0.44±0.44 | 0 | 0.396 |
| Malarial pigment | 1.63±0.71 | 4.00±0.00 | 0 | 0.007** |
| Lung macrophages | 1.75±0.25 | 2.89±0.26 | 1.00±0.00 | 0.011** |
| Inflammatory cell infiltration | 2.38±0.32 | 2.11±0.26 | 1.00±0.00 | 0.609 |
| Lung injury score | 12.75±0.75 | 19.67±0.88 | 6.17±0.17 | 0.001** |

*p*-values*- comparison between non-PE and PE groups

******Significant difference of *p* < 0.05
